# “Pretty Women” and “Lucky Blokes”: Unpacking Australian Social Media Responses to Female-Perpetrated Sexual Assault Against Men

**DOI:** 10.1177/08862605241239446

**Published:** 2024-03-17

**Authors:** April Murphy, Andrew Groves

**Affiliations:** 1 Deakin University, Burwood, VIC, Australia; 2 Flinders University, Adelaide, SA, Australia

**Keywords:** social media, female sex offender, sexual violence, male victim-survivor, female pariah

## Abstract

Female-perpetrated sexual violence research in Australia and elsewhere has been limited, part of a less common and arguably contentious field of criminology. Because of gendered social and cultural stereotypes, female sexual offending is often perceived as harmless or too rare to warrant attention. Utilizing Schippers’ pariah femininities, this paper presents a critical criminological exploration of social media users’ constructions of female sex offenders and their male victim-survivors. Examining 28 Facebook posts from 13 popular Australian newspapers, our findings identified social media users’ tendency to question offence severity and sexualize offenders based on appearance, revealing how offender legitimacy and conceptions of harm are shaped by gendered expectations of “pretty women” and “lucky blokes.” Conclusions suggest online discourse remains influenced by gendered stereotypes, though awareness of pariah femininities is growing, with further research needed worldwide to explore the impact of such social media attitudes and commentary on the incidence of and reactions to female sexual offending against men, including victim-survivors’ help-seeking behavior.

## Introduction

Despite growing interest in female-perpetrated sexual violence, significant gaps remain in critical criminological literature on the harms, impacts, and responses to female sex offenders and their male victim-survivors (Cortoni et al., 2017; Loxton & Groves, 2021; Walker et al., 2020). Within Australia, sexual violence is an enduring social and criminological issue characterized by widespread under-reporting, where what is known is far outweighed by what is not. Sexual violence is a deeply gendered issue, affecting 86% of women and 14% of men in Australia (Australian Bureau of Statistics [ABS], 2020). The infrequent reporting of female-perpetrated sexual violence has allowed rape myths to flourish creating falsehoods that it is less harmful than when male-perpetrated (Allen-Collinson, 2009; Fisher & Pina, 2013; Stathopoulos, 2014; Smith et al., 1988; Walker et al., 2020). It is often considered counterintuitive, rare, and thus ignored, or reframed and excused through traditional gendered lenses, where the notion that a woman could overpower a bigger, stronger man is viewed as unrealistic or impossible (Gölge et al., 2021; Hall, 2016; Hayes & Carpenter, 2013; Fisher & Pina, 2013). Over time, these myths have grown in online spaces, altering social discourses regarding how, when, where and who can experience and perpetrate sexual violence.

Recent advocacy and public awareness surrounding sexual violence in Australia can be attributed to the growing momentum of social movements and online activism, featuring alongside and shaped by critical scholarship. Movements like #metoo, #ibelieveher, #whyididntreport and #march4justice have highlighted the powerful role social media plays when seeking to increase cultural awareness and foster change (PettyJohn et al., 2019). Though sexual violence activism is not limited to online approaches, the ability to spread awareness, gain support and organize physical protests is a feature of social media’s functionality (Komazec & Farmer, 2020; PettyJohn et al., 2019). At the time of data collection, 84% of Australians used the internet daily, with a similar proportion (79%) engaging social media (Sensis, 2017). Given the “hyper-connectivity” (Wood, 2017) made possible by virtual networking, social media plays a key role in disseminating social commentary on important events (Komazec & Farmer, 2020; Weathers et al., 2016). However, an individual’s experience with and interpretation of online social discourse can depend on their interaction with popular platforms (Nikolov et al., 2018; Thumlert et al., 2022), such as Twitter, Facebook, Instagram, and TikTok (among others). Personalized algorithms or “filter bubbles” (Nikolov et al., 2018) have come to define online navigation, creating and curating relevant content for users based on their preferences, values, and biases (Nikolov et al., 2018; Thumlert et al., 2022). Because of this, users are more likely to be exposed to and engage with content that supports their worldview. This “bubbling” means social media data cannot be considered indicative of all Australian social media users; however, it does offer valuable insight into the influence of online personal bias on social discourse. As users can share their beliefs, biases, attitudes, and assumptions with relatively few consequences, commentary may rely on individual prejudices and misinformation without any form of balance. This is particularly problematic for victim-survivors of sexual violence who face victim-blaming, rape myths, and scrutiny over the integrity of their reporting (Komazec & Farmer, 2020; Nagy, 2017; Stubbs-Richardson et al., 2018).

The current paper evaluates these perspectives in the context of Australian Facebook users’ commentary on articles of female-perpetrated sexual violence, building upon previous scholarship (see Loxton & Groves, 2021). It builds upon the limited Australian perspectives of female-perpetrated sexual violence, viewing such offending through the lenses of Schippers’ (2007) female pariah and sexism as a tool of patriarchy (see Bryson, 2021). While earlier research examined online attitudes toward male victim-survivors (of female sex offending), this paper focuses on the nature and extent of social media responses to the female perpetrators of sexual violence. Looking from the other side of this offender-victim dichotomy, we articulate shifts in online commentary directed at female sex offenders, indicative of a gendered discourse characterized by “pretty women” and “lucky blokes.” The following section explores the extant literature on female sex offenders, focusing on the concepts of “luck” and Schippers’ (2007) female pariah, contextualizing the current online climate and response to female sex offenders. Utilizing a mixed-method approach comprising thematic, content and discourse analyses, the paper then presents an interrogation of social media commentary from 28 Facebook posts, discussing cases of female sex offending presented in 13 different Australian newspapers. Our conclusions discuss future directions and illustrate the increasing value of fostering critical scholarship regarding female sex offenders.

## Background

### Offending Rates

Female sexual offending represents an emerging field of criminological interest, a product of its relative infrequency and limited reporting (Beech et al., 2009). As sexual violence is primarily perpetrated by men against women, there has been less demand for research addressing the harms caused by female sex offenders (Almond et al., 2015; Hayes & Baker, 2014; Stathopoulos, 2014; Walker et al., 2020; Walklate et al., 2022). Though impacted by significant underreporting, an estimated 3% to 5% of sexual offences are committed by women, which includes violence against children, men, and other women (Almond et al., 2015; Stathopoulos, 2014; Tidmarsh & Hamilton, 2020). Unpacking these figures, Cortoni et al. (2017) collected global survey data from 2006 to 2013, which revealed that although female sex offending represents a small percentage of police reports and court cases (approximately 2%), a much larger proportion is not reported (12%) within the criminal justice system (CJS). Additionally, female sex offenders were more inclined to offend against men (40%) than women (4%) (Cortoni et al., 2017).

A notable feature of Cortoni et al.’s (2017) research was its distinction between official and unofficial reporting, where informal sharing and social recognition within the community was more likely, while the CJS was perceived as much slower to recognize female sex offenders. For example, in reviewing legal responses to female sex offenders, Bickart et al. (2019) noted that 2,017 reports of sexual assault by women in Queensland often resulted in “cautions or diversions.” Similar judicial responses have been observed across Australia, where from 2019 to 2020, 8% (*n* = 21) of women accused of sexual assault were acquitted, and 23% (*n* = 60) of cases were withdrawn by the prosecution (Australian Bureau of Statistics (ABS), 2021). Such legal responses prevent greater recognition of this issue by limiting attention to individual cases and constraining or obscuring estimates of prevalence, which some scholars have argued has led to greater leniency (Jeffries & Bond, 2010), reinforcing gender stereotypes. Given the “dark figure” of female-perpetrated sexual violence against men, further critical criminological interrogation of this and other forms of female sex offending is warranted.

### The Paradox of “Luck” and Rape Myths

The notion of “luck” in sexual violence has historically been tied to notions of masculinity, where sex with an older woman represents a “rite of passage.” Angelides’ (2010) analysis of female-teacher sexual interactions with students illustrated common media narratives for particularly attractive female offenders who were overtly sexualized for their physical appearance. Because of these perceptions, online media and social discourses presented similar responses to female paedophiles and sex offenders, strengthening “lucky bastard” narratives (Angelides, 2010; Zack et al., 2018). Historically, responses to female sex offenders have reflected gender stereotypes that generalize women to traditional roles of “non-violent, non-sexual, caregivers” (Almond et al., 2015) or “fragile nurturers” (Loxton & Groves, 2021). Because of this, women are rarely considered capable of committing intentional harm. Turton (2010) explores such social mores, drawing attention to how some responses to paedophilic mothers sought to reframe their abuse as a form of maternal instinct, where lines between caregiving and harm were blurry at best. Hayes and Carpenter (2013) speak to this blurriness, suggesting that reliance on misinformed understandings of “motherly love” can lead to social discourse that questions the extent of female-perpetrated harm, a question not asked in experiences of male-perpetrated violence.

Similar disparities were evident in Smith et al.’s (1988) foundational study assessing US college students’ misconceptions of sexual violence through fictional mixed-sex and same-sex vignettes. Student responses indicated more punitive attitudes towards male sex offenders than female sex offenders and believed that male victim-survivors would derive “more pleasure” from female sexual assault. These findings represent some of the early scholarship that evidenced common rape myths suggesting sexual violence perpetrated by women against men is “less traumatic” (Smith et al., 1988; Struckman-Johnson & Struckman-Johnson, 1992). This historical research indicates a systematic rejection of female sex offending and its associated harms, instead viewing such violence as a sexual fantasy or “desirable” outcome (Loxton & Groves, 2021). Such perspectives emphasize the paradox of luck, as articulated by one participant from Smith et al.’s (1988) study who wrote, “Some guys have all the luck!”

### The Female Pariah

Schippers’ (2007) conceptualization of the female pariah represents an important shift to more feminist understandings of crime, deviance, and offending. Hegemonic femininity is characterized by “womanly” traits that frame and legitimize a hierarchical and complementary relationship to Connell and Messerschmidt’s (2005) hegemonic masculinity. Hegemonic masculinity is widely accepted as the positioning of dominant characteristics that reflect social expectations of “real men” such as stoicism and aggression, which overwhelm “subordinate” characteristics like compassion, empathy, and the desire to nurture (Connell & Messerschmidt, 2005; Walklate et al., 2022). In critiquing hegemonic femininity and constructing the female pariah, Schippers (2007) sought to present a theory of femininity that explored social responses to conforming or deviant female behaviour. As Schippers (2007) describes, hegemonic femininity is a “compliance to the patriarchy,” relying on characteristics that define women in traditional languages, such as “warm,” “virtuous,” and “nurturing.” Hegemonic femininity, therefore, works to maintain the putative “balance” between masculinity and femininity, guaranteeing the dominant position of men over others (not just women) (Connell & Messerschmidt, 2005; Schippers, 2007). In this context and in line with the wider feminist literature (Bryson, 2021), it is important then to critically unpack the concept of patriarchy to avoid generalist notions, as these typically minimize women’s experiences, overlooking the nuances—for example—of female offending. These characteristics demonstrate the need to instead use the language of sexism as a tool of patriarchy, as one component of a complex and intractable system that continues to hinder recognition of women who sexually offend, as doing so would recognize their “offending” behaviors as serious, harmful, and intentional. To wit, Schippers (2007) argued that should a woman embody and practice masculine behaviors, such as dominance, aggression—or sexual violence—they disrupt this balance. These masculine portrayals “contaminate” and threaten “men’s exclusive possession of hegemonic masculine characteristics,” referred to as pariah femininities (Darwin, 2017; Schippers, 2007). Notably, this concept also incorporates evaluation of the attendant processes of demonization and stigmatization that occur when a woman embodies masculine qualities and challenges the integrity of what it means to “be a man,” capturing both individual characteristics of the woman and the social framing inherent in others’ or community reactions when perceiving/constructing them as a pariah.

Exploring the influence of these pariah femininities, Hayes and Baker (2014) assessed notions of female innocence and tangible harm by observing how female sex offenders are presented in Australian and UK news media. Their analyses revealed that female sex offenders were downplayed in online print media, where most articles reproduced traditional gender characterizations, like “mother” and “wife” (Hayes & Baker, 2014). Similarly, Starr’s (2022) study of women bartenders found that when women step into typically masculine work roles, they become pariahs, where they are fetishized to ensure they “remained feminized workers in masculine workplaces” and “render[ed]. . . objects under patriarchy.” We argue Starr’s (2022) notion of pariah femininities must also include its criminological representation to capture female sex offenders and the tendency to sexualize them even in the context of deviance as an attempt to return them to passive, sexual conquests. Starr’s (2022) discussion helps to frame how female sex offenders depart from anticipated feminine behaviors. The perpetration of intentional harm and aggression again threatens socialized and patriarchal concepts of masculine and feminine roles. In contrast, Christensen’s (2018) analysis of female child sex offenders depicted in Western print mediafound offenders can be masculinized and constructed as dangerous. In their sample, 31 newspaper reports directly addressed the psychological harms and severity of offenders’ abuse, omitting sympathetic labels like “mother.” These findings reflect non-traditional reactions in certain media, suggesting the possible construction of and/or reference to pariah femininities and acknowledgment of the harms associated with female sex offenders, though Christensen (2018) emphasized the need for further exploration in online spaces. In the current study then, application of the female pariah lens was examined through social media users’ framings of female offenders’ characteristics, behaviors, and roles (as related to men), which shaped the coding of key themes and analysis of the data.

As the use of online/digital technologies increases as a feature of everyday social life, greater recognition is needed of how people engage with, discuss, and respond to key social issues (including their perception and treatment of others). In response to this lacuna, Bhuptani et al. (2023) explored the impact of sexual violence disclosures in online spaces after #MeToo, examining correlations between online social reactions and Post-traumatic Stress Disorder (PTSD). Their work outlined that negative responses to online disclosures of sexual violence were associated with heightened risks of PTSD, demonstrating the detrimental impact social media can have on victim-survivors if experiences are not subsequently recognized and/or validated. Considering these responses to victim-survivors, Bhuptani et al. (2023) suggested that greater research is needed into understanding the contribution of online social reactions to disclosures. This is central to understanding and/or explaining experiences of serious crime and victimization, as well as the social and CJS responses to them (Ammerman & Jones, 2020; Stathopoulos, 2014). In breaking free of these patriarchal ideologies or criminologies, this paper examines attitudes towards women who engage in sexual violence against adult men, answering Connell and Messerschmidt’s (2005) enduring call for greater interrogation of femininities. Our analysis helps to increase understanding of female sex offenders by building upon the limited Australian perspectives of female-perpetrated sexual violence (Stathopoulos, 2014; Walker et al., 2020). Our findings confirm the female pariah as a valuable, albeit complicated lens through which to view social reactions to female-perpetrated sexual violence, particularly when gender scripts diverge from traditional expectations.

## Methods and Materials

This research examined the content and narratives embedded in Facebook users’ comments posted to news articles regarding female-perpetrated sexual violence across several popular Australian newspapers’ Facebook pages (see Loxton & Groves, 2021).^1^ The authors examined perceptions of and reactions to female-perpetrated sexual violence against men. Specifically, whether such commentary reflects social reinforcement of traditional characterizations shaped by hegemonic femininities/masculinities (i.e., men as offenders; women as victim-survivors) or the emergence of pariah femininities.

Facebook is a popular, global social media platform for the sharing of digital information and opinions, upon which users can also “post” social commentary in real time (Edosomwan et al., 2011). In 2019, almost two-thirds of Australians reported regularly accessing or engaging with Facebook (Cowling, 2019; Napoleoncat, 2019). Facebook also provides user-friendly search and filtering tools for its pages and posts, expediting data collection compared with other popular social media platforms (e.g., Twitter) and rationalizing its research utility. In terms of “data,” Facebook posts typically comprise an uploaded image, comment, or article (e.g., link to news report), on which other users can comment and/or react (e.g., “like”) (Herring, 2004). Enabled by the Internet, Facebook is a globally accessible medium whereby users can access and comment on any “public” pages or posts, regardless of geographic location or boundaries. To try to isolate Australian users’ responses, this study purposively sampled popular Australian newspapers, to capture and enable evaluation of local social commentary (Davies et al., 2011; Walter, 2013).

The research sample comprised 28 Facebook posts from 13 online Australian newspapers published across 2009 to 2019, each of which had established a Facebook page by 2009, allowing development of a substantial online “following.” The final sample is outlined in Table 1, representing the number of posts sourced from each newspaper and their level of popularity (e.g., Facebook page “like” and followers). The number of “likes” for individual comments has been included with users’ qualitative text, demonstrating the embeddedness of these narratives. Newspapers that did not have a Facebook page or at least 10,000 followers were excluded from the sample, recognizing their limited engagement or user commentary.

**Table 1.** Facebook Newspaper and Post Source. Includes Page “Likes” and “Followers.”

Facebook Newspaper Page	No. of Posts in Sample	No. of Page Likes*	No. of Page Followers*
The Geelong Advertiser	1	71,374	71,549
The Australian Financial Review	1	107,496	113,101
The Age	2	307,046	309,755
The West Australian	1	308,520	312,412
Northern Territory News	3	339,758	330,630
The Advertiser	3	362,297	354,940
The Herald Sun	1	363,190	359,256
Perth Now	2	449,210	439,123
The Courier Mail (QLD)	5	507,583	488,479
The Sydney Morning Herald	1	1,114,180	1,099,146
News.com.au	5	1,117,954	1,157,667
The Daily Telegraph (NSW)	1	1,323,934	1,301,950
The Daily Mail Australia	2	4,068,847	4,573,606

* Number of Facebook page ‘likes’, and followers recorded on June 21, 2019.

From 10 to 21 June 2019, 27 Australian newspapers were examined, of which 13 operated linked Facebook pages. Table 2 details the final sample of 28 Facebook news posts drawn from these 13 pages. Posts ranged from 2014 to 2019, discussing Australian cases (*n* = 8) and international media reports of sexual violence (*n* = 20). The sample was generated using search terms: “male rape victims,” “male victim,” “female sex offender,” and “male sexual assault victim.” To be included, posts needed to contain an adult male victim-survivor of female-perpetrated sexual violence, have more than 10 comments, not discuss child sexual assault or same-sex victimization, and have been posted from 2009 to 2019. Once included, the comment section of each post was searched to obtain a raw sample of 2,561 user comments. As Facebook’s comment tracking feature is inaccurate (including comments removed/deleted later, which is common practice), all comments were manually counted. Comments posted in a language other than English, duplicates, “spam,” or those that only included “tags” to other users without comment were excluded, producing a final sample of 1,621 comments.

**Table 2.** Final Data Sample Including News Title, Associated Paper, and Date.

Newspaper Source	Post Number	Total Comment Sample	Total Clean Sample
The Sydney Morning Herald	Post: #1	138	43
The Age	Post: #2; #3	247	107
Daily Telegraph	Post: #4;	178	104
The Courier Mail	Post: #5; #9; #11; #12; #20	324	212
The Advertiser	Post: #6; #21; #22	293	172
The Australian Financial Review	Post: #7	21	10
The West Australian	Post: #8	89	63
Perth Now	Post: #10; #23	172	104
The Northern Territory News	Post: #13; #15; #16	133	109
Geelong Advertiser	Post: #14	35	19
Daily Mail Australia	Post: #17; #19	187	112
News.com.au	Post: #18; #24; #25; #26; #28	644	495
The Herald Sun	Post: #27	100	71
	Total	2,561	1,621

**Image 1.**
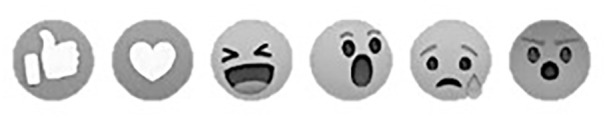
Original Facebook reactions in 2016. *Source.* Loxton and Groves (2021).

**Image 2.**
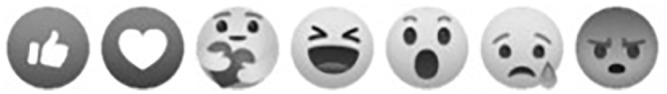
Updated reactions of 2020. *Source.* Loxton and Groves (2021).

As the study assessed users’ overall engagement (similar to Ksiazek et al., 2016), posts were also analyzed quantitatively through users’ “reacts”; a feature introduced by Facebook in 2017, with six ways to interact with posts—“like,” “heart,” “laugh,” “shock/wow,” “sad,” and “angry” (see Image 1). These were updated in 2020, adding a “care” option (see Image 2).

**Graph 1.**
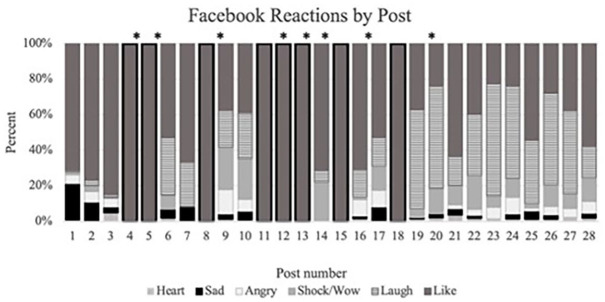
Facebook reactions per post. *Source.* Loxton and Groves (2021). *Posts published prior to 2016 did not have the option of “Facebook reacts.”

Graph 1 presents the rate of reactions per post (except those published before 2016, which did not have the “reacts” option, identified by asterixis). Other than “likes,” which were the most common reaction, Graph 1 reveals how many responses to posts of sexual violence involved “laughs”—post 19 (56%), post 20 (58%), post 23 (63%), and post 24 (52%)—a seemingly imprudent response that emphasizes the need to examine users’ attitudes toward male victim-survivors or female-perpetrated sexual violence.

### Research Procedure

Our study used an inductive mixed method, engaging quantitative and qualitative analyses of publicly available Facebook comments. A content and discourse analysis of users’ comments and “reacts” assisted in categorizing and organizing the language employed by Facebook users (Davies et al., 2011; Krippendorff, 2004). Use of a primarily qualitative approach revealed central themes, stories, and opinions across all 28 posts. Key variables included type of response (i.e., textual or “react” responses), and whether it accepted/supported or denied/criticized the notion of the female sex offender. Though most comments were text based, some were images or “GIFs” (a moving image), a small number of which were retained, as their meaning and intention were unambiguous. Once coded, posts were categorized thematically, using inductive analysis to guide interpretation of users’ responses. Braun and Clarke’s (2006, 2021) six-step thematic analysis was used to identify prominent themes, helping to characterize this online milieu. These processes were valuable in identifying and unpacking the sociocultural value of rape myths, pariah femininities, and offender sexualization in line with extant literature (Krippendorff, 2004).

Distilling a range of emerging themes, seven female-offender specific codes were identified among 1,621 comments. *Sexualization of the offender/offence* was the most common form of commentary, with users seeking to restructure abuse as a fantasy or fetish. *Crime de-escalation* worked in tandem with sexualization of the offence/offender, as comments relied on rape myths to downplay harms experienced. *Offender appearance* regarded commentary that suggested someone considered attractive was not capable of causing harm. *Rejection of female sex offenders*, similarly, played into traditional scripts of femininity, with comments denying women’s capacity for harm. Users who acknowledged *Double standards* highlighted the unequal social and legal treatment of female sex offenders compared with their male counterparts. Some users also sought to *Dispel rape myths*, using the opportunity to challenge gendered stereotypes of rape and sexual violence. Users who *Acknowledged female sex offenders* represented a growing corpus who recognize the harms caused by female-perpetration of sexual violence and voiced desire for punishment.^2^

### Limitations

Given the global reach and accessibility of Facebook that render physical borders meaningless, users’ engagement and profiles cannot be considered exclusive to Australia (Loxton & Groves, 2021). Furthermore, although the authors filtered the sample by popular Australian media sources, online profiles may not contain authentic information allowing users to falsify or fabricate data, where lack of regulatory oversight means comments must be considered *prima facie*. Additionally, the biases created by personalized algorithms and filter bubbles mean that commentary obtained from these posts cannot reflect broader public opinions, limiting the generalizability of the data. However, it offers a snapshot of online social discourses and responses to female sex offenders and their male victim-survivors.

Secondly, it is acknowledged that personal biases may have influenced the development and reporting of the study. However, content and discourse analyses are subjective tools by nature, whereby interpretation will be iteratively unique (Walter, 2013). As noted above, the research employed a detailed process of triangulation, comprising evaluation of relevant theory, method, and investigator oversight (see Denzin, 1978; Noble & Heale, 2019). In line with the broader literature, this process enabled the authors to garner thematic findings from the content and discourse analyses and explore the data on a deeper level, to gain a more extensive understanding of online responses to male victimization. Finally, we have offered transparent and detailed descriptions of all processes to further combat bias.

## Findings

Analysis of Facebook users’ comments and “reacts” to Australian articles of female sexual violence against men revealed several common themes, including discussions of “lucky” experiences and responses to attractive offenders, amongst growing social awareness. Drawing on the intersections between gender values and social media characteristics, this study sought to discuss what factors shape Facebook users’ perceptions of female sex offenders and how these might reflect or challenge pariah femininities (Schippers, 2007). The findings should be considered in line with the above discussion of the balance between hegemonic masculinities and femininities and how the female pariah challenges the accepted, or at least expected gendered norms that subjugate women. Specifically, although the paper focuses on perceptions of female sex offenders, much of the commentary remains rooted in hegemony, presented in terms of what it means for men, which we explore further in the Discussion.

### The “Lucky” Bloke’s Perspective

*Hyper-sexualized Fetishes*: Sexualized language was found extensively throughout all 28 Facebook posts, though the nature of the commentary varied. Several users overtly placed the offender in a sexual position, while others expressed desire to experience similar “treatment” (*n* = 20), believing the victim-survivor was a “lucky bloke. . .lol” (comment from post #14). Specifically, 231 users (14%) sexualized female offending, and a further 118 (7%) directly discussed offenders’ attractiveness or a victim-survivors’ “lucky break.” These responses represent a lack of maturity around male sexual victimization and recognition of the harms of female-perpetrated sexual violence. For example, in relation to the victimization of a male taxi-cab driver (post #9), some users noted:

Clearly, I’m in the wrong job! :P

*(comment from post #9)*

*Likes*: 3

*Applies to be a cab driver

*(comment from post #9)*

*Likes*: 11

These comments illustrate a disconnect between harm and fantasy, as users appeared to focus on the sexual aspects of the act and what they could (or would like to) benefit/receive from the interaction rather than its impact as a serious crime. The sexualization of these women reinforces rape myths that position men as only those capable of committing serious sexual crimes (Hayes & Carpenter, 2013). These comments are consistent with the acceptance of hegemonic femininities, where women are not recognized for the harm they cause and are instead reverted to passive characters through sexualization (Hayes & Carpenter, 2013; Schippers, 2007). One user demonstrated the extent of their offender’s sexualization and fantasy, de-valuing the woman’s actions by asking:

How is this a crime. I wish someone would hold a knife to my throat while a girl performed sexual acts on me, steal $32? Lol. Cheaper than a date where you don’t get laid

*(comment from post #12)*

*Likes*: 57

Remarkably, comments that displayed similarly overt sexualization or reinforced hegemonic understandings of gender roles (e.g., marriage, possession, etc.) received considerable support and “likes” from other users:

She sounds like a keeper

*(comment from post #4)*

*Likes*: 43

What’s her number? I’ll marry her!

*(comment from post #5)*

*Likes*: 32

Though subtle, this represents the online reinforcement of social and cultural norms, which seek to reassert the masculine/feminine balance (Schippers, 2007), constructed in the language and terms of the subjugation of women. In doing so, these findings validate extant research that suggests such negative, but traditional comments are more common than those that display genuine concern (Stubbs-Richardson et al., 2018).

*Harm-minimizing*: Facebook users minimized the impacts of female sex offending by shifting focus away from individual incidents and experiences. Specifically, users delegitimized its “criminality” and either justified offenders’ actions or used statistical evidence to illuminate the “insignificance” of the offending. Crime de-escalation was consistent across all 28 posts, evident in broad debates regarding feminism or expressions of disbelief. Other efforts were more explicit, where the lower rate of female offending was used to minimize harm:

How can a female rape a man, it has always bewildered me.

*(comment from post #2)*

*Sad reacts*: 1

. . . Women harassing men is the lowest category . . . you know who’s harassing men? OTHER MEN . . . the problem is STILL men.

*(comment from post #1)*

*Likes*: 3

Though acknowledging the predominance of male sexual violence offending, the comment effectively delegitimizes all victim-survivors’ experiences (Andersen, 2011), strengthening common rape myths that ultimately disadvantage both men and women. Adherence to these hegemonic views was reinforced by claims such as:

“In 12 months, we arrested 172 sex offenders—three have been female. The proportion is very different, we are interested in what drives this to happen”. Three in 172? Those female offenders are OUT OF CONTROL.

*(comment from post #18)*

*Likes*: 7

Others stated that male rape was “. . .weird” (comment from post #14) or that it could occur “. . .only in America” (comment from post #13), simultaneously reinforcing rape myths and de-escalation. This created a duality in the meaning of “lucky”: where it is unlikely to happen, but if it does, men should enjoy it.

### “Pretty” (but not) Harmful?

When female-perpetrated sexual assault occurs, traditional gender stereotypes hinder its social acknowledgment, as such events deviate from expected feminine behavior or sexual violence scripts (Schippers, 2007; Turton, 2010). Our findings suggest that users’ sexualization of offenders’ physical appearance ultimately questions female pariahs’ capacity to cause harm when they are attractive (Schippers, 2007).

*Offender Appearance*: Consistent with prior research (Zack et al., 2018), many users (*n* = 98) excused female sex offenders’ behavior based on physical appearance, ignoring the potential for deliberate harm. In one article (post #16) concerning a conventionally attractive US “teacher-lover” case with an 18-year-old male victim-survivor, online commentary hyper-sexualized the female teacher, suggesting “teachers have changed. For the better” (comment from post #15). Physical appearance was used to excuse offender behavior, where it neutralized women’s perceived accountability.

I have left my window open for years, never had

that sort of luck, she looks ok.

*(comment from post #14)*

*Likes*: 14

The queue of men lined up to marry this woman

starts in Sydney and ends in Perth!

*(comment from post #7)*

*Likes*: 2

**Image 3.**
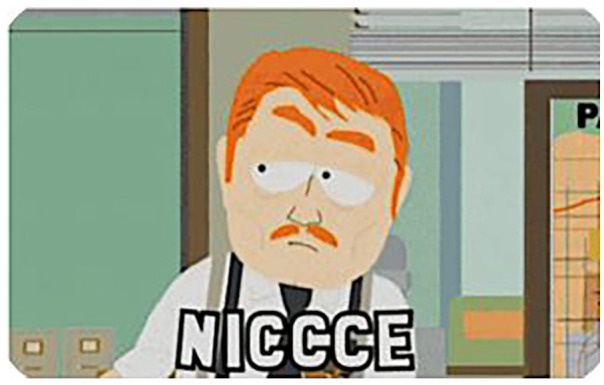
Social media comment.

*(comment from post #27)*

*Likes*: 21

Physical attractiveness also influenced comments concerning victim-survivors’ lack of response to their victimization, further neutralizing offenders’ behavior. Posts #12 and #13 both described cases of female sex offenders; however, the latter more strongly emphasized the woman’s weight, with a user subsequently commenting that “being raped by a fat chick is horrific enough” (comment from post #13). Conversely, comments from post #12 questioned victim-survivors’ lack of enjoyment, where the woman was considered more attractive. Several justified the offender’s actions implying “he [the victim] obviously is gay! Or she was hideous! Otherwise, what’s the problem?” (Comment from post #12). The overt sexualization, reinforcement of heteronormative stereotypes, and assessments of physical attractiveness collectively contributed to the acceptance of pariah femininities and the denial of harm. Reinforcing Darwin’s (2017, p. 20) representation of the “fat pariah,” which conflates worth with physical appearance, female sexual violence against men is inconceivable when the offender is “pretty” but becomes possible (though still not automatically accepted) when she is not.

*Offender Capacity*: Consistent with the prior literature, online users struggled to consider the harms committed by women as equal to those committed by men, or indeed possible at all (Christensen, 2018; Zack et al., 2018). Users’ disbelief was evident in comments such as “mental illness is at play here” (comment from post #11) or “I call BS” (comment from post #18). Subtle forms of evaluative commentary that rejected women’s capacity for sexual harm were also observed in comments that again viewed women’s actions through a hegemonic masculinity lens, where it was noted that “women aren’t perfect. . . but they aren’t the gender responsible for the vast majority of violence. . .” (comment from post #3). While acknowledging the gendered nature of sexual violence, these responses devalued the impact of this behavior, while shifting the blame away from women (Stathopoulos, 2014). Such responses have serious consequences for male victim-survivors affecting the likelihood of reporting and the perceived legitimacy of victimhood (by victims and the community). Others quantified the disproportionate experience of female sexual violence against men:

. . . Look mate, all assault is bad, but as soon as women start

murdering men at the rate of one per week then we’ll talk, ok?

*(comment from post #3)*

*Likes*: 2

Oh boo hoo!! I don’t condone any violence of any kind toward males or females . . . but WTF!? When there is a report of woman after woman day after day being killed at the hands of men or their partners, I don’t think posting a story about a very small amount of attacks on men by women is appropriate . . . to say the least!

*(comment from post #18)*

*Likes*: 2

These responses served two functions: firstly, to reinforce perceptions of women’s limited capacity to cause harm through sex, maintaining stereotypical expectations of the balance between masculinity and femininity. Secondly, they promote reactive rather than proactive approaches to female sex offending, using hegemonic femininity as a barrier to greater recognition and debate. Collectively, these effectively gatekeep what it means to be a “victim,” which is counterintuitive and detrimental to all victims and victims’ movements (Andersen, 2011). This commentary reveals a tendency for social discourse to rely on easy and convenient (hegemonic) stereotypes when attributing blame. These limit the recognition of pariah femininities, inhibiting meaningful discussion and reform of criminal justice policy and practice, despite being attached to a news article discussing graphic female sex offending.

### Growing Awareness

Despite overwhelming rejection and/or sexualization of female sex offenders, some awareness, and acknowledgment of male victim-survivors’ trauma was evident. Several online users presented a contrasting profile from the previously discussed themes, as those who recognized the harms caused by female sex offenders were more inclined to reject traditional expectations and hegemonic femininities (Schippers, 2007). These comments emphasized a heightened awareness of female sex offending, with almost one-fifth (17%, *n* = 281) of users acknowledging the damage caused by gendered double standards and more than one-quarter (26% or *n* = 415) expressing concern for victim-survivors.

*Addressing Double Standards*: As noted in the method, users’ online commentary was coded into two separate sub-themes, “social double standards,” and “legal double standards.” Social double standards were most recognized among online users with one-fifth (17% or *n* = 281) understanding that men and women have vastly different experiences with responses to sexual violence, aptly captured in the statement that:

When a man gets raped, everybody laughs and cracks jokes. But if a woman gets raped then everyone is all serious about it. Rape is rape.

*(comment from post #13)*

*Likes*: 59

These comments represented an unexpected shift in response to female sex offenders, emphasizing the traumatic impact of sexual offending—regardless of the offender’s gender (Allen-Collinson, 2009). Similarly, comments highlighting the social and legal double standards received higher levels of support through increased number of “likes,” as demonstrated in the following comments:

Imagine if it were the other way around? Would we all think it was funny then? Why is it so funny when a female perpetrator assaults a male? Until our attitudes change, there will never be equal rights

*(comment from post #5)*

*Likes*: 37

Male or female, NO means NO

*Likes*: 162

*Reply to thread. . .*

Agreed. I hope she learns her lesson. If it had been a man acting that way against a woman, there would be all kinds of people up in arms. Because it’s the other way around doesn’t mean that it’s okay. It isn’t.

*(comments from post #8)*

*Likes*: 19

Perceptions of legal double standards, however, were less progressive, with 45 comments discussing the legal dispensations given to female sex offenders, such as lighter outcomes. Users recognized that “Accusations seem to be enough to remove a man from his job—what exactly is needed for a [woman]??” (Comment from post #7), exemplifying users’ recognition of pariah behaviors but also an acknowledgment of the wider impacts.

*Acknowledging Harm*: Users’ acknowledgement of female-perpetrated sexual violence was another emerging theme, where a small proportion of users avoided sexualizing offenders and instead viewed such behavior as distressing and harmful. One user identified that “Women also sexually harass males. [It is] not just a single gender issue” (comment from post #2). Another more graphically stated:

That poor man. He is brave for coming forward, I really hope they catch those sick bitches, to stop them from hurting anyone else

*(comment from post #17)*

*Likes*: 36

These responses emphasize a shift in online social responses to perpetrators and victim-survivors of sexual violence and the historical acceptance of rape myths. Others displayed more acute awareness of female sex offenders, dropping preconceived or sexualized notions and engaging in more negative discursive descriptions of perpetrators’ actions as “disgusting behavior” (comment from post #10). This commentary offered nuanced recognition of pariah behaviors, illustrating how gender does not necessarily govern victim-survivors’ experiences of sexual violence but can acutely affect experiences of related social reactions.

## Discussion

The definition and position of the female sex offender in social discourse is contentious. Our findings suggest many social media users seek to defend patriarchal or hegemonic views of women’s sexual behavior. In dismissing women’s capacity for sexual violence, Facebook users in this study revealed a nuanced construction of the female pariah, with users’ attitudes predominantly concerned with, and shaped by, negative perceptions of the characteristics, behaviors, and roles of these offending women, while others recognized the need for greater criminological attention on this type of offending.

We examined the ways in which social media users’ commentary engaged with the notion of pariah femininities in relation to female sexual victimization of men. Our findings revealed that online responses hyper-sexualized some female sex offenders, who were then viewed as attractive, with their harms minimized to fit stereotypical, hegemonic values, reframing violence experienced by men merely as sexual advances by “pretty women.” In turn, these experiences were commonly constructed as socially desirable, with victim-survivors paradoxically labelled as “lucky blokes.” Other responses characterized female sex offending as a form of “sexual deviance” aligned with Schipper’s (2007) categories of the “slut” or sexually aggressive “badass girls,” which at once diminish the seriousness of these contexts of violence and represent outsider, patriarchal (read sexist) labels. Such labelling suggests an inherent duality where men are looking in and commenting on other men’s experiences of sexual victimization perpetrated by women, yet who arguably lack the shared cultural understanding and experience that women have traditionally endured, while simultaneously their commentary serves to “other” these women as outsiders (Becker, 1963). Again, the construction of these women as pariahs was mitigated by traditional expectations where their actions were not perceived as dangerous. As supported by prior literature and in line with the framing of sexism as a tool of patriarchy (Bryson, 2021), our findings revealed that online users deconstructed female sex offenders, returning many of the women to passive, subjugated positions, ultimately removing their agency (Hayes & Baker, 2014; Hayes & Carpenter, 2013; Schippers, 2007; Starr, 2022; Zack et al., 2018). Sexism, in this way, acts as a tool of the patriarchy that strips female pariahs of power and control, even in events where women are engaging in harm or deviance. As seen in online commentary, women were continually re-framed as sexual objects and conquests, with little to no recognition of the intentional, serious, and harmful behavior they perpetrated. The presence of news reports on female-perpetrated sexual violence against men is deeply threatening to wider notions of male sexual authority and wider social dominance, which may further explain the continued reframing of female offenders as sexual objects, as “phallic drift” (see Bryson, 2021). In such cases, online responses redirected the discourse to evaluate the “problem” from men’s perspectives and sought to blame men for failing to view their abuse/assault positively. These responses reflect a modern application of Angelides’ (2010) “lucky bastard,” allowing commenters to not only justify sexual violence (by women, against men), but restructure it as a “rite of passage.” This represents a substantial barrier to recognition of female sexual violence against men that must be overcome if responses to either female perpetrators or male victims are to be effective.

The online commentary similarly evidences Darwin’s (2017) pariah femininity hierarchy, where offenders are reduced to stereotypical labels and subjugated, albeit in varying positions. As again demonstrated above, this sample of female sexual offenders have been considered and constructed, in both language and status, as they relate to the position and perceptions of men. Users repeatedly commented on women’s appearance and behavior, conflating these characteristics with the capacity to cause harm with only those considered unattractive and behaving inappropriately perceived as dangerous. This further demonstrates the harmful impacts of negative social reactions in online spaces, whereby online responses that reject the capacity of female sexual offenders to cause harm can negatively influence recovery for victim-survivors and potentially threaten their help-seeking behavior Bhuptani et al. (2023). Notably, this hierarchy was extended beyond critiques of women where in the commentary on post #12, the man’s sexuality was questioned as part of an ‘if not (unattractive), then (you must be homosexual)’ binary explanation for why they (the victim) identified the behavior as violent assault. Essentially, this commentary further distances women’s intent and agency from notions of harm or trauma, constructing female pariahs but only in certain contexts. Instead, most of the women were subjugated to positions of inferiority and powerlessness and not perceived as *real* threats—as pariahs in name only, limiting appropriate responses to both female offenders and their victims.

Another feature of this milieu was discussion of the rarity of female-perpetrated sexual violence, a “fact” wielded by commenters to minimize the impact of individual experiences and overall harms caused by women against men. These comments revealed that online users believe the negative connotations associated with sexual offending are lessened or removed when gender roles are reversed, emphasizing the complexity of the female pariah characterization, and the inherent “confusion” or denial that occurs when attitudes remain influenced by traditional or normative views (see Hayes & Carpenter, 2013). Specifically, it reflects a social complacency toward female sexual offenders and the need for greater awareness of this social phenomenon, but simultaneously the targeted rejection of male victim-survivors, strengthening previous findings (see Loxton & Groves, 2021).

Although many participants dismissed female sexual offending by certain types of women, some revealed awareness of the harms caused, demonstrated through careful unpacking of social and legal double standards. On one hand, many online users described a perceived leniency towards female sex offenders, where punishment is largely symbolic or heavily reduced when compared with male sex offenders. On the other hand, social double standards were more diverse in their presentation, where some users were seemingly unconcerned, demonstrated through the increased use of humor or “laugh” reacts to news posts. Conversely, other comments addressing double standards described a more genuine acceptance of pariah femininities, with online users looking beyond traditional excuses of “contamination,” and instead recognizing the intention and deep-seated harm that can be caused by female-perpetrated sexual violence against men. Notably, within this sample of Facebook users, some acknowledged the need for greater understanding of the unique features and consequences of female sex offending. Compared with previous scholarship (Christensen, 2018), several users recognized the challenges (and benefits) for men in coming forward, which illustrates growing interest in attending to female-perpetrated sexual violence against men and the need for further research in this field.

These findings support conclusions raised by Bhuptani et al. (2023) and justify greater critique of media reporting and better understanding of social discourses surrounding sexual violence. This sample of users’ commentary provides insight into the existence and consequences of filter bubbles of online bias regarding cases of female-perpetrated sexual violence against men, emphasizing the need for mechanisms by which information and counter-narratives can be shared to offer more balanced perspectives. There is evidence here that social commentary reflects normative sociocultural perceptions of female sex offenders as rare and largely harmless. There is need, then, for greater evidence-based campaigning and education about sexual violence, building of e-safety principles and practices, as well as regulation of the quality/accuracy of media reporting (see Bhuptani et al., 2023; Landor & Eisenchlas, 2012). Recognition of female sex offending may also help to foster deeper evaluation of social media harms against victim-survivors, and the impact it may have on their willingness to come forward or seek help. Lastly, our findings rationalize a rethinking within critical criminology to push beyond hegemonic conceptualizations of sexual violence where offending and victimhood are considered analogous with masculinity and femininity, respectively (see Walklate et al., 2022). Employing a feminist epistemology necessitates building profiles of women’s offending, including female sex offenders, using perspectives like Schippers’ (2007) female pariah that challenge existing values and norms rather than rely on understandings of women’s behavior viewed through patriarchal, hegemonic lenses.

## Conclusion

Examining 28 Facebook posts discussing female-perpetrated sexual violence against men from 13 different Australian newspapers, we revealed complex online discourses and attitudes surrounding sexual violence and social media users’ reliance on hegemonic, gender normative categorizations of offenders and victims. Social media comments challenged purist application of Schippers’ (2007) pariah femininities, with users seeking to sexualize, fetishize, and minimize women’s capacity to cause harm, which fostered a culture of denial toward the threat of so-called pariahs, except in certain contexts (where women were not attractive). Instead, online users revealed a tendency to reject female sex offenders, subjugating them to positions of inferiority, perceived as pariahs in name only, not as real threats. Such discourses regarding female-perpetrated sexual violence against men arguably constrain practices of reporting and efforts to seek support for men and limit how the justice system, friends, families, and wider communities manage the reality of female sex offenders. We argue there is need for a holistic response that is more preventative (through education) and fosters accountability and action for change through increased awareness and quality of social discourse (through building of e-safety principles and media regulation). Such a response would logically include further research into social attitudes or responses to female-perpetrated sexual violence against children, same-sex or LGBTQIA+ communities, and intimate partners, which although outside the scope of this paper are important cohorts in this milieu. Though sexual violence continues to be principally perpetrated by men, from a critical criminological perspective scholars must recognize the harms and impacts of female-perpetrated sexual violence. Doing so will not only strengthen feminist literature and improve victim-survivor support services and associated public policy and practice, both in Australia and internationally, but also recognize the need for efforts to reduce broad social complacency toward female sexual offenders and increase awareness of this social phenomenon.

## Notes

1. This method corresponds with the approach employed in a previous paper, which focused on the experiences of male victim-survivors of female-perpetrated sexual violence (see Loxton & Groves, 2021). Data draws from the same project but engages different themes and concepts to build new knowledge of female sex offenders—the other side of this offender-victim dichotomy.

2. Comments responding to males’ experiences of acts of sexual victimization have been critically examined in previous research (see Loxton & Groves, 2021).
